# Supplementary material for: Tofacitinib Treatment Suppresses CD4+ T-Cell Activation and Th1 Response, Contributing to Protection against Staphylococcal Toxic Shock
Source: Int J Mol Sci. 2024 Jul 7;25(13):7456. doi: 10.3390/ijms25137456 (PMC11242597; doi:10.3390/ijms25137456)
Supplement: Supplementary file 1 [file ijms-25-07456-s001.zip › ijms-3059923-supplementary.pdf]

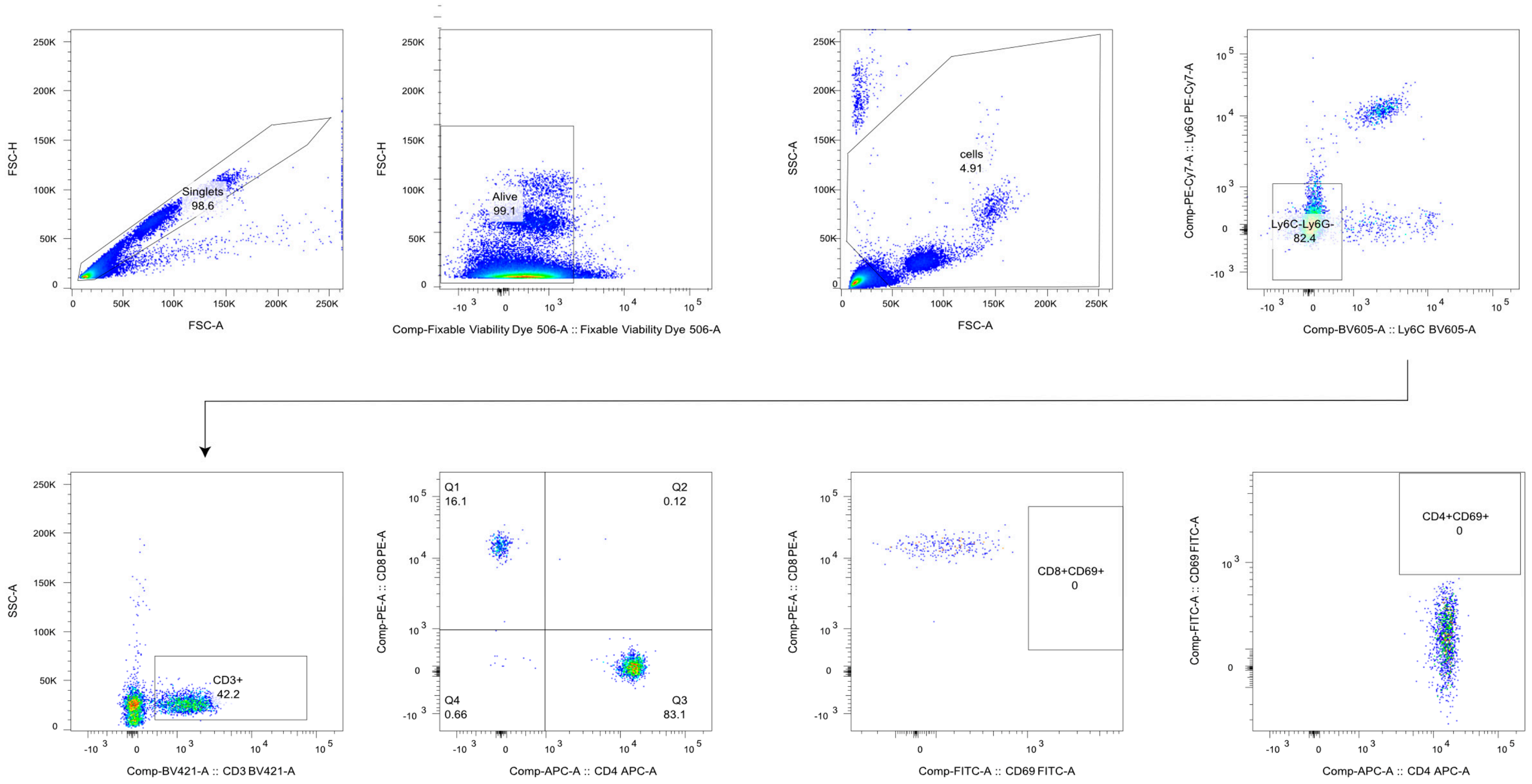

Supplementary Figure S1. Representative gating strategy of FACS experiments. Balb/c mice were divided into groups given either tofacitinib (n=9) or vehicle only as control (n=9) and then challenged with toxins. Untreated mice (n=5) that were not given any toxins or treatment were used for reference. Antibodies used were BV421-conjugated anti-CD3 (BioLegend, San Diego, California, USA), PerCP-Cy5.5-conjugated anti-CD3 (BioLegend), APC-conjugated anti-CD4 (BioLegend), PE-conjugated anti-CD8a (eBioscience, San Diego, California, U.S.), FITC-conjugated anti-CD69 (Invitrogen), PE-Cy7-conjugated anti-Ly6G (BD Bioscience), BV605-conjugated anti-Ly6C (BD Bioscience) and Fixable viability dye 780 (eBioscience). The anti-Ly6C and anti-Ly6G were used to exclude neutrophils and monocytes before gating for CD3+ for improved precision.
